# Supplementary material for: Genomic instability caused by Arp2/3 complex inactivation results in micronucleus biogenesis and cellular senescence
Source: PLoS Genet. 2023 Jan 27;19(1):e1010045. doi: 10.1371/journal.pgen.1010045 (PMC9907832; doi:10.1371/journal.pgen.1010045)
Supplement: S1 Table — (PDF) [file pgen.1010045.s001.pdf]

**S1 Table. Oligonucleotides.**		
**RT-PCR Primers**		
β-actin	F	CCGTGAAAAGATGACCCAGATCATG
	R	ATGCCACAGGATTCCATACCCAAG
Cdkn1a	F	TGATGTCCGACCTGTTCCGCAC
	R	GCCCACCCGGGGAATCTTCA
Gapdh	F	ATTGTGGAAGGGCTCATGACCAC
	R	GTAGCCGTATTCATTGTCATACCAGG
Interleukin-6	F	AAGTTCCTCTCTGCAAGAGACTTC
	R	GAGGAAATTTTCAATAGGCAAATTTCCTG
Interferon-Beta	F	ACTATAAGCAGCTCCAGCTCCAAG
	R	AAGTGGAGAGCAGTTGAGGACATC
**Cloning Primers**		
cGAS	F	ATCATCGGTACCATGGAAGATCCGCGTAGAAGGA
	R	ATCATCGCGGCCGCTCAAAGCTTGTCAAAAATTGGAAACCC
**siRNAs**		
siControl		(Sigma #SIC001)
siCdkn1a[A]		CAGACCAGCCUGACAGAUU
siCdkn1a[B]		GCCUUGUCGCUGUCUUGCA
