# Supplementary material for: Genomic instability caused by Arp2/3 complex inactivation results in micronucleus biogenesis and cellular senescence
Source: PLoS Genet. 2023 Jan 27;19(1):e1010045. doi: 10.1371/journal.pgen.1010045 (PMC9907832; doi:10.1371/journal.pgen.1010045)
Supplement: S2 Table — (PDF) [file pgen.1010045.s002.pdf]

S2 Table. Immunofluorescence and Immunoblotting Reagents.				
Target	Probe	Animal	Conc.	Identifier
**Primary Antibodies (Immunofluorescence)**				
Arp3	anti-Arp3	Mouse	1:350	Sigma (A5979)
ArpC2	anti-ArpC2/p34-Arc	Rabbit	1:500	Millipore (07-227-I)
cGAS	anti-cGAS	Rabbit	1:1,000	Proteintech (26416-1-AP)
γH2AX	anti-Phospho-Histone H2A.X	Rabbit	1:750	Cell Signaling (9718)
GM130	anti-GM130	Mouse	1:2,000	BD Biosciences (610822)
GRP94	anti-GRP94	Rat	1:1,000	Novus (NB300-619)
Lamin B1	anti-Lamin B1	Rabbit	1:250	Proteintech (12987-1-AP)
IRF3	anti-IRF3	Rabbit	1:250	Cell Signaling (4302)
p21	anti-p21	Rabbit	1:500	Proteintech (28248-1-AP)
p53	anti-p53	Rabbit	1:1,000	Proteintech (10442-1-AP)
P-p53	anti-Phospho-p53(S15)	Rabbit	1:500	Cell Signaling (9284)
STING	anti-STING	Rabbit	1:1,000	Proteintech (19851-1-AP)
P-STING	anti-Phospho-STING (S365)	Rabbit	1:250	Cell Signaling (72971)
Tubulin	anti-Beta-Tubulin	Mouse	1:10,000	DSHB (E7)
**Primary Antibodies (Immunoblotting)**				
Actin	anti-Beta-Actin	Mouse	1:5,000	Proteintech (66009-1-Ig)
Arp3	anti-Arp3	Mouse	1:1,000	Sigma (A5979)
ArpC2	anti-ArpC2/p34-Arc	Rabbit	1:1,000	Millipore (07-227-I)
cGAS	anti-cGAS	Rabbit	1:500	Proteintech (26416-1-AP)
GAPDH	anti-GAPDH	Mouse	1:20,000	Proteintech (60004-1-Ig)
Il-1-Beta	anti-Il-1-Beta	Hmstr	1:500	eBioscience (14-7012)
Lamin B1	anti-Lamin B1	Rabbit	1:500	Proteintech (12987-1-AP)
mCherry	anti-mCherry	Rabbit	1:1,000	Abcam (ab167453)
p53	anti-p53	Rabbit	1:1,000	Proteintech (10442-1-AP)
P-p53	anti-Phospho-p53(S15)	Rabbit	1:1,000	Cell Signaling (9284)
STING	anti-STING	Rabbit	1:1,000	Proteintech (19851-1-AP)
Tubulin	anti-Beta-Tubulin	Mouse	1:10,000	DSHB (E7)
**Secondary Antibodies (Immunofluorescence)**				
Mouse IgG	Alexa 555, 647 anti-Mouse	Goat	4 ug/ml	Life Technologies (A21424, A21236)
Rabbit IgG	Alexa 488, 555 anti-Rabbit	Goat	4 ug/ml	Life Technologies (A11034, A21429)
Rat IgG	Alexa 488 anti-Rat	Goat	4ug/ml	Life Technologies (A11006)
**Secondary Antibodies (Immunoblotting)**				
Rabbit IgG	HRP anti-Rabbit	Donkey	1:10,000	GE Healthcare (NA934V)
Mouse IgG	IRDye 680 anti-Mouse	Donkey	0.05 ug/ml	LI-COR (926-32212)
Rabbit IgG	IRDye 800 anti-Rabbit	Donkey	0.05 ug/ml	LI-COR (926-32213)
**Molecular Probes (Fluorescence)**				
DNA	4',6-diamidino-2-phenylindole (DAPI)		1 ug/ml	Invitrogen (D1306)
F-actin	Alexa 488-Phalloidin		0.2 U/ml	Invitrogen (A12379)
F-actin	Alexa 647-Phalloidin		0.4 U/ml	Invitrogen (A22287)
Lysosomes	Lysotracker Red		100nM	Invitrogen (L7528)
