# Supplementary figures and images for: Genomic instability caused by Arp2/3 complex inactivation results in micronucleus biogenesis and cellular senescence
Source: PLoS Genet. 2023 Jan 27;19(1):e1010045. doi: 10.1371/journal.pgen.1010045 (PMC9907832; doi:10.1371/journal.pgen.1010045)

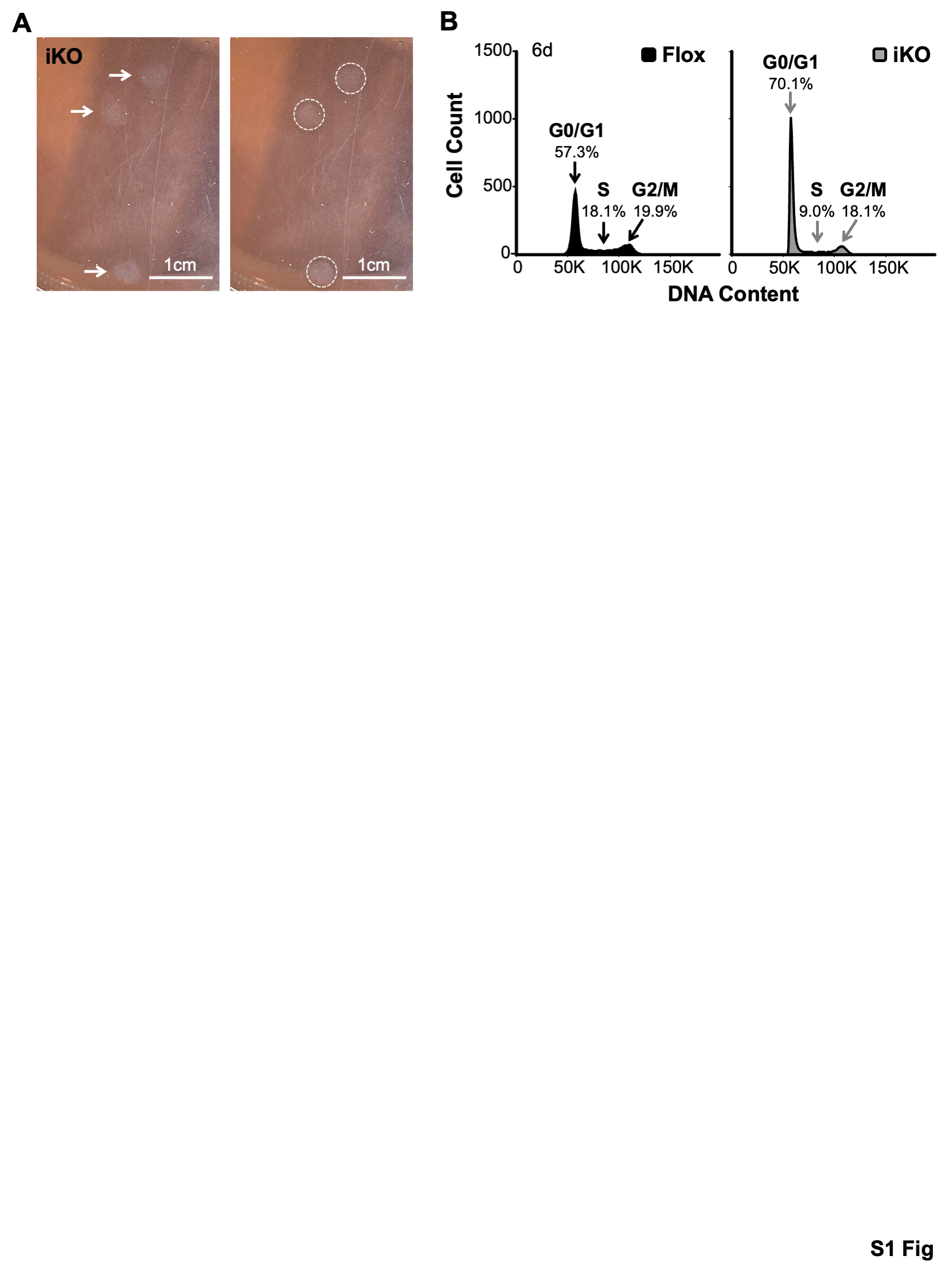

Supplement: S1 Fig — (A) Arpc2-floxed mouse tail fibroblasts (MTFs) were treated with DMSO (Flox) or 4-OHT (iKO) for 6d, transferred to a 6cm dish containing drug-free media, and examined by light microscopy using an iPhone at 12d. Arrowheads (left panel) and circular outlines (right panel) highlight colonies that either avoided CreER-mediated recombination at the Arpc2 locus, converted to a functional Arpc2-expressing variant, or acquired suppressors of the Arpc2 deletion. Colony formation was observed for approximately 1 out of every 100,000 cells. (B) Arpc2-floxed MTFs were treated with DMSO (Flox) or 4-OHT (iKO) for 6d, collected, fixed, stained with propidium iodide, and analyzed by flow cytometry. 10,000 events were examined for each cell type. The % of cells in each phase of the cell cycle was quantified using FlowJo software. (TIF) [file pgen.1010045.s003.tif]

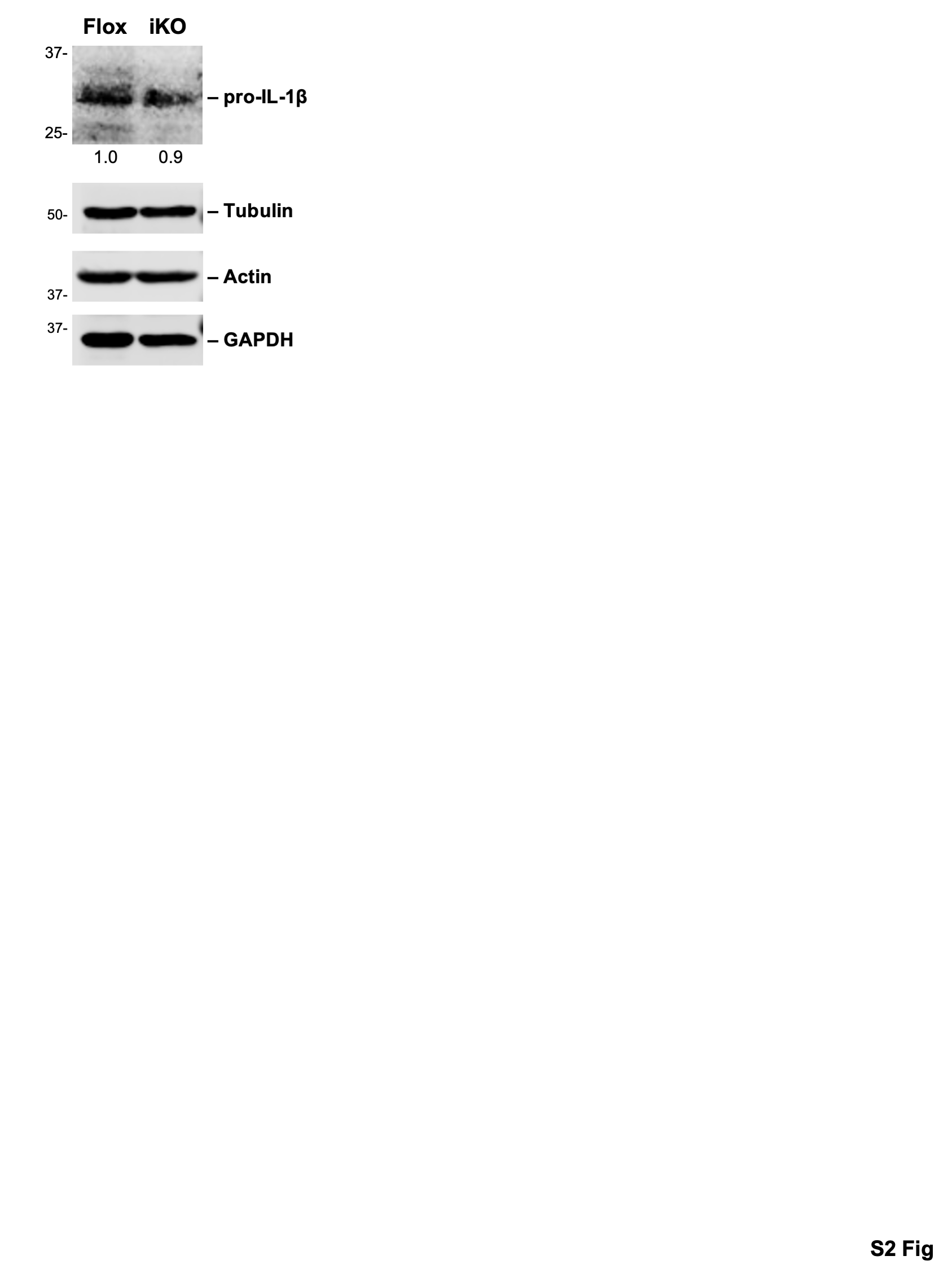

Supplement: S2 Fig — Mouse fibroblasts were treated with DMSO (Flox) or 4-OHT (iKO) for 6d and collected at 9d. Samples were lysed, subjected to SDS-PAGE, and immunoblotted with antibodies to IL-1β, tubulin, actin, and GAPDH. Pro-IL-1β densitometry values are shown. Cleaved IL-1β was not detected. (TIF) [file pgen.1010045.s004.tif]

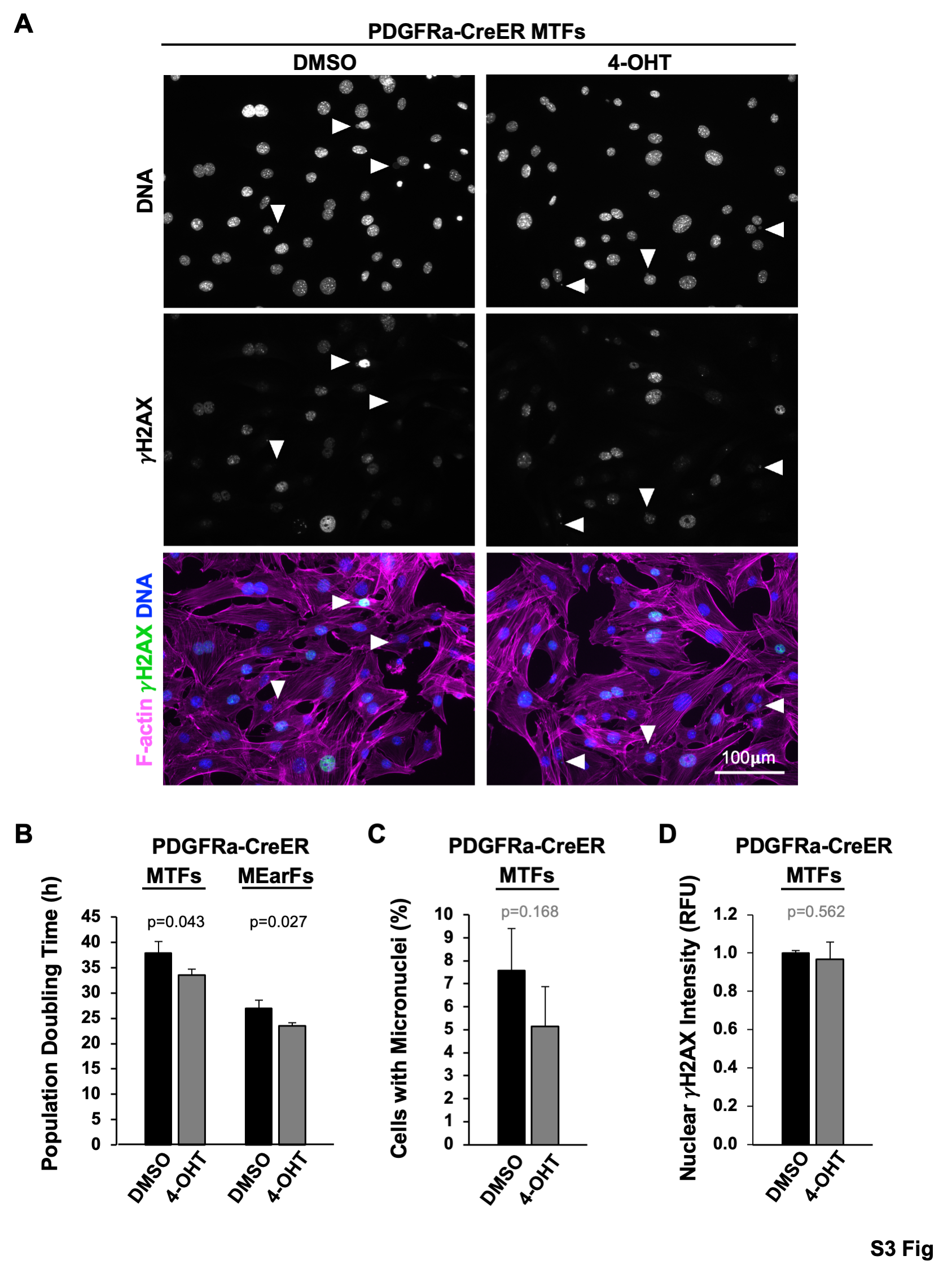

Supplement: S3 Fig — (A) Mouse tail fibroblasts (MTFs) with Platelet Derived Growth Factor Receptor alpha promoter-driven expression of a Cre recombinase fused to a human estrogen receptor ligand binding domain (PDGFRa-CreER) were treated with DMSO or 4-OHT for 3d, fixed, and stained with phalloidin (F-actin; magenta), a γH2AX antibody (green), and DAPI (DNA; blue). Arrowheads point to micronuclei. (B) PDGFRa-CreER MTFs or PDGFRa-CreER mouse ear fibroblasts (MEarFs) were treated with DMSO or 4-OHT for 6d, and population doubling times were quantified from 8-10d. Each bar represents the mean doubling time ±SD from n = 3 experiments. 4-OHT-treated cells replicated faster, not slower, than DMSO-treated control cells. (C) The % of PDGFRa-CreER MTFs with micronuclei was quantified following a 3d exposure to DMSO or 4-OHT. Each bar represents the mean % ±SD from n = 3 experiments (812–899 cells per bar). (D) Nuclear γH2AX levels were quantified by outlining the DAPI-stained nucleus of each cell in ImageJ and measuring the mean γH2AX pixel intensity. Each bar represents the mean % ±SD from n = 3 experiments. RFU = Relative Fluorescence Units. (TIF) [file pgen.1010045.s005.tif]

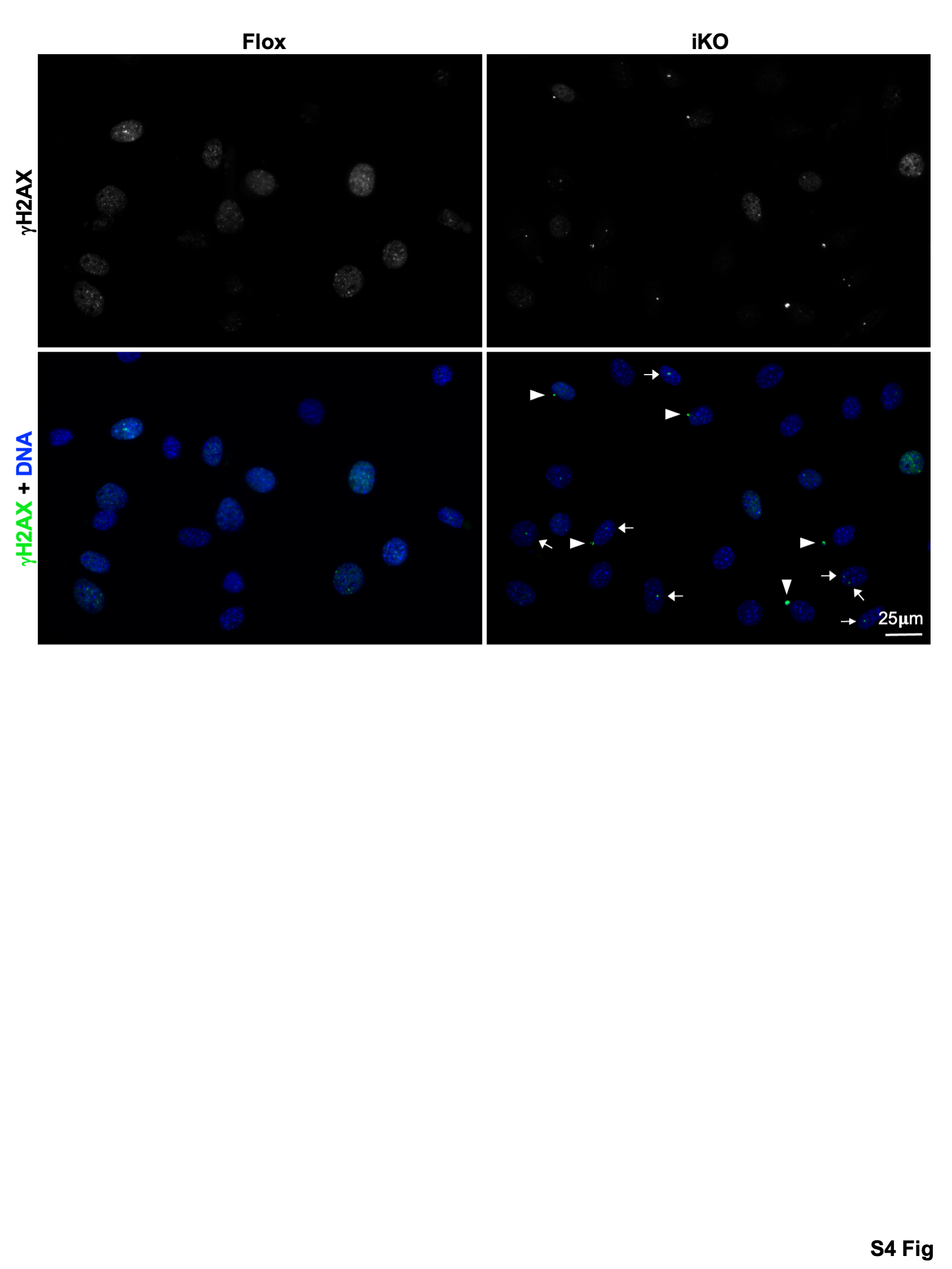

Supplement: S4 Fig — Mouse fibroblasts were treated with DMSO (Flox) or 4-OHT (iKO) for 6d, fixed at 7d, and stained with a γH2AX antibody (green) and DAPI (DNA; blue). Arrowheads point to γH2AX clusters in micronuclei and arrows indicate γH2AX clusters in nuclei. (TIF) [file pgen.1010045.s006.tif]

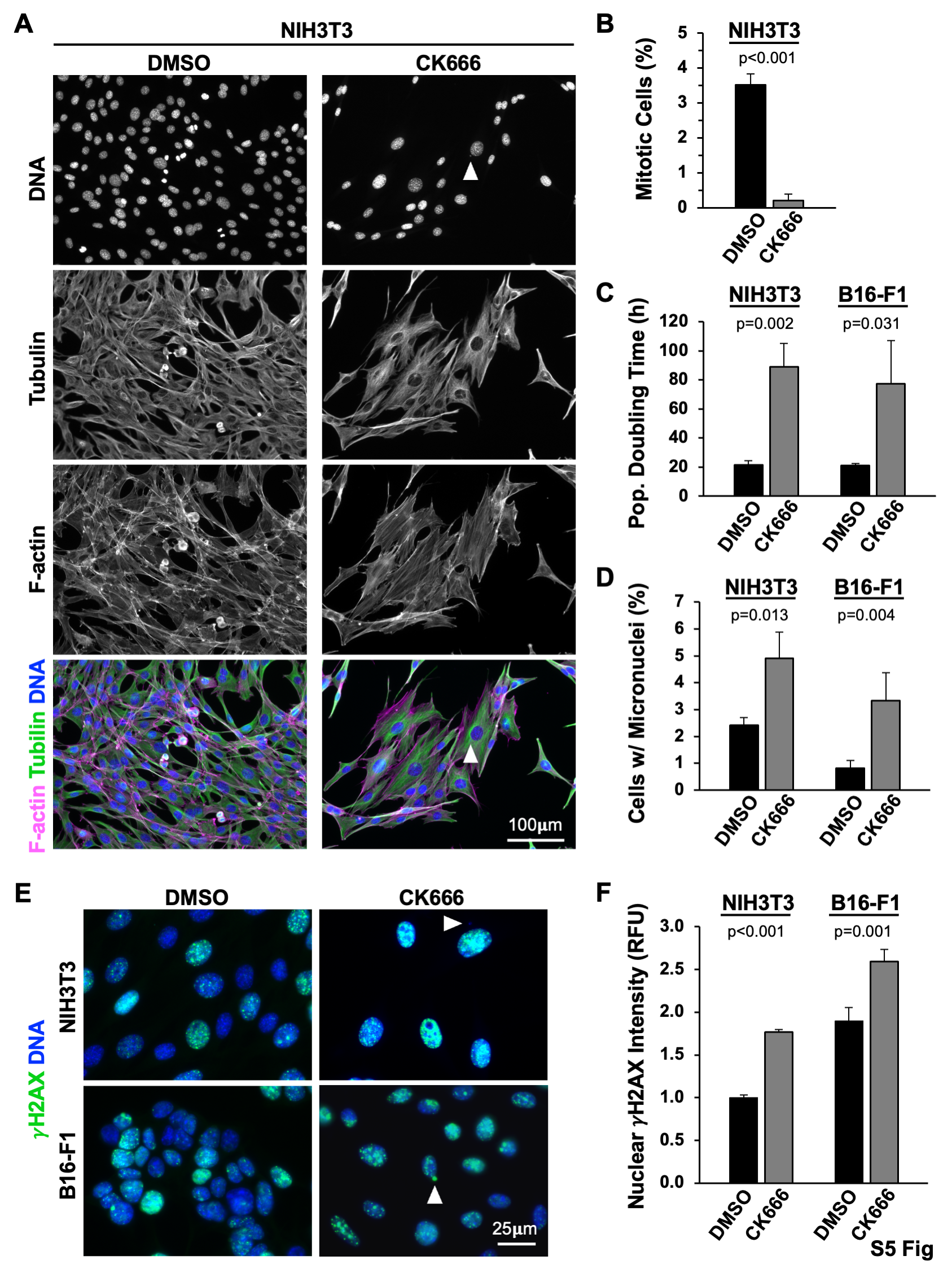

Supplement: S5 Fig — (A) NIH3T3 and B16-F1 cells were treated with DMSO or 100μM CK666 for 35h (with DMSO or CK666 media changes at 0h, 12h, and 24h), fixed, and stained with phalloidin (F-actin; magenta), an anti-tubulin antibody (green), and DAPI (DNA; blue). Only NIH3T3 cells are shown. Arrowheads highlight micronuclei. (B) The % of NIH3T3 cells in mitosis was quantified for samples treated as in A. Each bar represents the mean % ±SD from n = 3 experiments (999–1684 cells per bar). (C) NIH3T3 and B16-F1 cells were treated as in A and population doubling times were quantified. Each bar represents the mean doubling time ±SD from n = 3 experiments. (D) NIH3T3 and B16-F1 cells were treated as in A and the % of cells with micronuclei was quantified. Each bar represents the mean % ±SD from n = 3 or 4 experiments (354–602 cells per bar). (E) NIH3T3 and B16-F1 cells were treated with DMSO or CK666, fixed, and stained with a γH2AX antibody (green) and DAPI. (F) Nuclear γH2AX levels were quantified by outlining the DAPI-stained nucleus of each cell in ImageJ and measuring the mean γH2AX pixel intensity. Each bar represents the mean % ±SD from n = 3 or 4 experiments (100–240 cells per bar). RFU = Relative Fluorescence Units. (TIF) [file pgen.1010045.s007.tif]

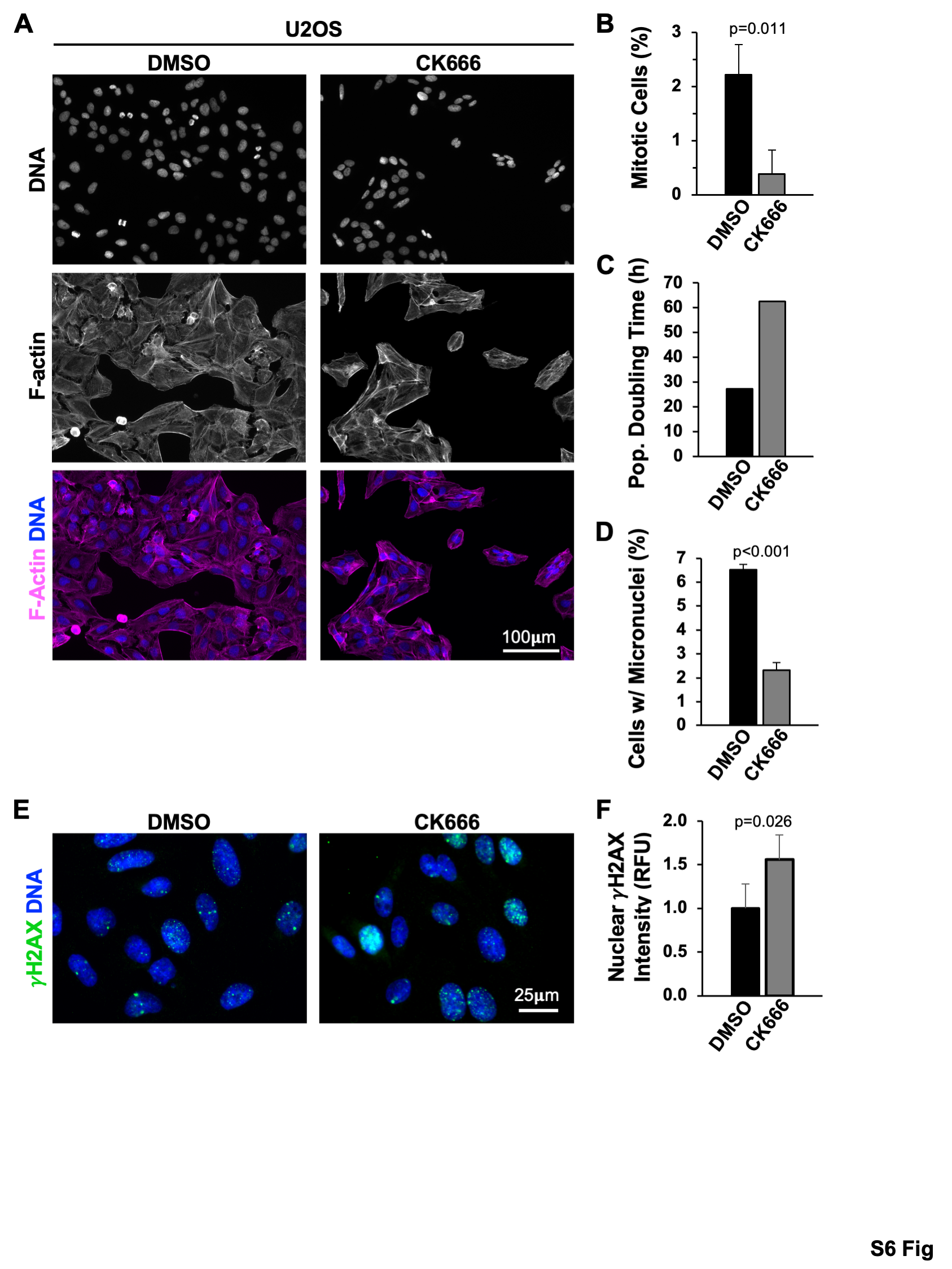

Supplement: S6 Fig — (A) U2OS osteosarcoma cells were treated with DMSO or 200μM CK666 for 60h (with DMSO or CK666 media changes at 0h, 12h, 24h, 36h, and 48h), fixed, and stained with phalloidin (F-actin; magenta) and DAPI (DNA; blue). (B) The % of U2OS cells in mitosis were quantified for samples treated as in A. Each bar represents the mean % ±SD from n = 3 experiments (1198–1454 cells per bar). (C) Cells were treated as in A and population doubling times were quantified. Each bar represents the doubling time from a representative experiment. (D) Cells were treated as in A and the % of cells with micronuclei was quantified. Each bar represents the mean % ±SD from n = 3 experiments (1198–1454 cells per bar). Surprisingly, CK666-treated cells had fewer micronuclei than DMSO-treated cells. (E) Cells were treated with DMSO or CK666, fixed, and stained with a γH2AX antibody (green) and DAPI. (F) Nuclear γH2AX levels were quantified by outlining the DAPI-stained nucleus of each cell in ImageJ and measuring the mean γH2AX pixel intensity. Each bar represents the mean % ±SD from n = 3 experiments (890–937 cells per bar). RFU = Relative Fluorescence Units. (TIF) [file pgen.1010045.s008.tif]

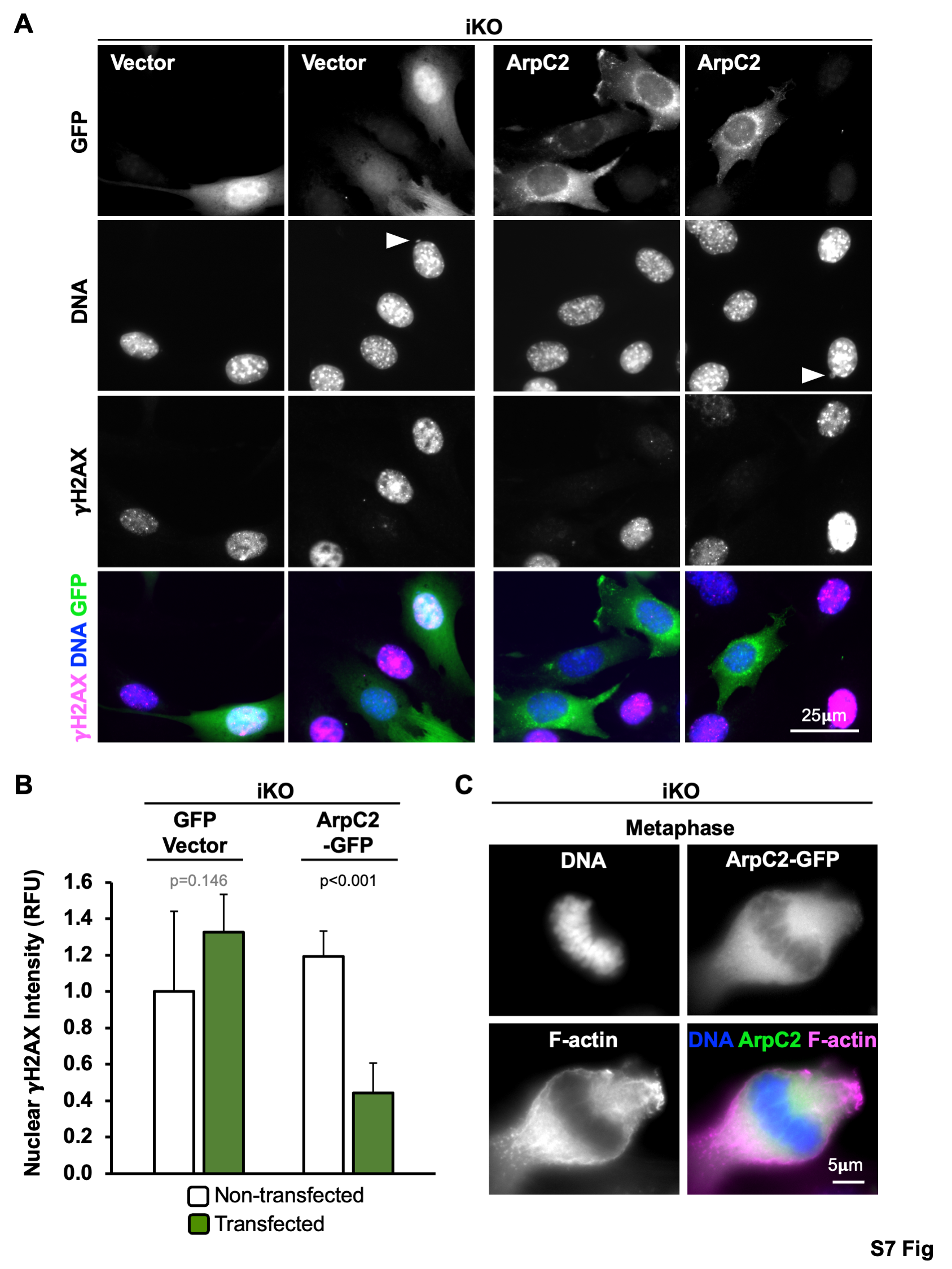

Supplement: S7 Fig — (A) Mouse fibroblasts were treated with 4-OHT (iKO), transfected with plasmids encoding GFP or ArpC2-GFP (green), fixed at 2d, and stained a γH2AX antibody (magenta) and DAPI (DNA; blue). Arrowheads point to micronuclei. (B) Nuclear γH2AX levels were quantified by outlining the DAPI-stained nucleus of each cell in ImageJ and measuring the mean γH2AX pixel intensity. Each bar represents the mean % ±SD from n = 12–24 cells per category. RFU = Relative Fluorescence Units. (C) Cells were transfected as in A and stained with phalloidin (F-actin; magenta). An ArpC2-GFP-expressing cell in metaphase is shown. Note the penetration of ArpC2-GFP and F-actin into the central chromatin mass. (TIF) [file pgen.1010045.s009.tif]

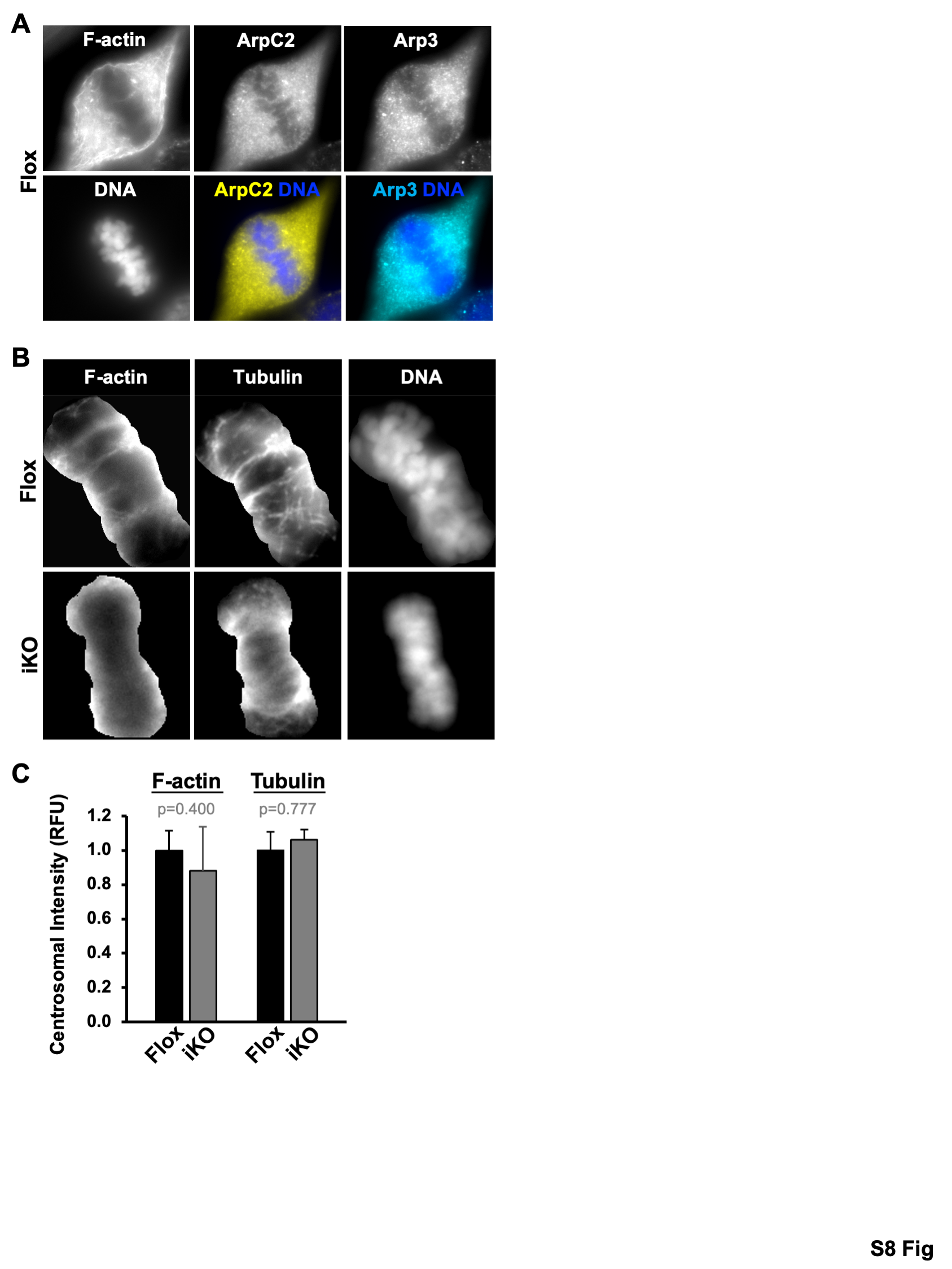

Supplement: S8 Fig — (A) Mouse fibroblasts (Flox) were treated with DMSO for 1-2d, fixed, and stained with phalloidin (F-actin), anti-ArpC2 antibodies (yellow), an anti-Arp3 antibody (cyan), and DAPI (DNA; blue) as in Fig 6A. (B) The DNA-containing region was isolated from representative spindles in Fig 6B and 6C and magnified. (C) Mouse fibroblasts were treated with DMSO for 1-2d, fixed, and stained with phalloidin, an anti-tubulin antibody, and DAPI as in Fig 6C. Circles of 5μm diameter were drawn around centrosomes in metaphase cells and the fluorescence intensities of F-actin and microtubules were measured as in Fig 6D. Each bar represents the mean intensity ±SD from n = 24 metaphase centrosome-associated regions compiled from 3 experiments. (TIF) [file pgen.1010045.s010.tif]

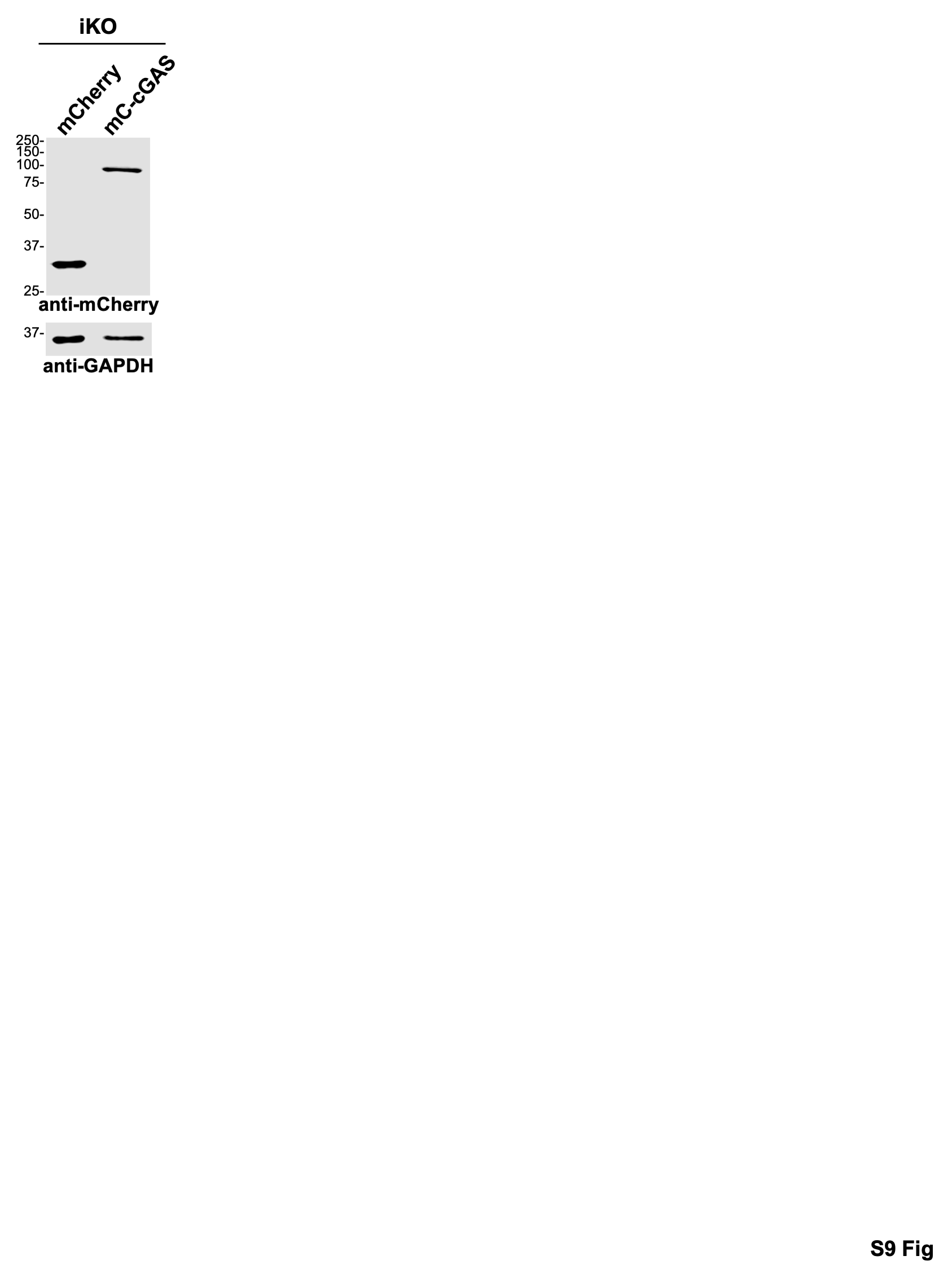

Supplement: S9 Fig — Mouse fibroblasts were treated with 4-OHT (iKO), transfected with plasmids encoding mCherry or mCherry-cGAS as in Fig 10A, collected, and immunoblotted with antibodies to mCherry and GAPDH. (TIF) [file pgen.1010045.s011.tif]

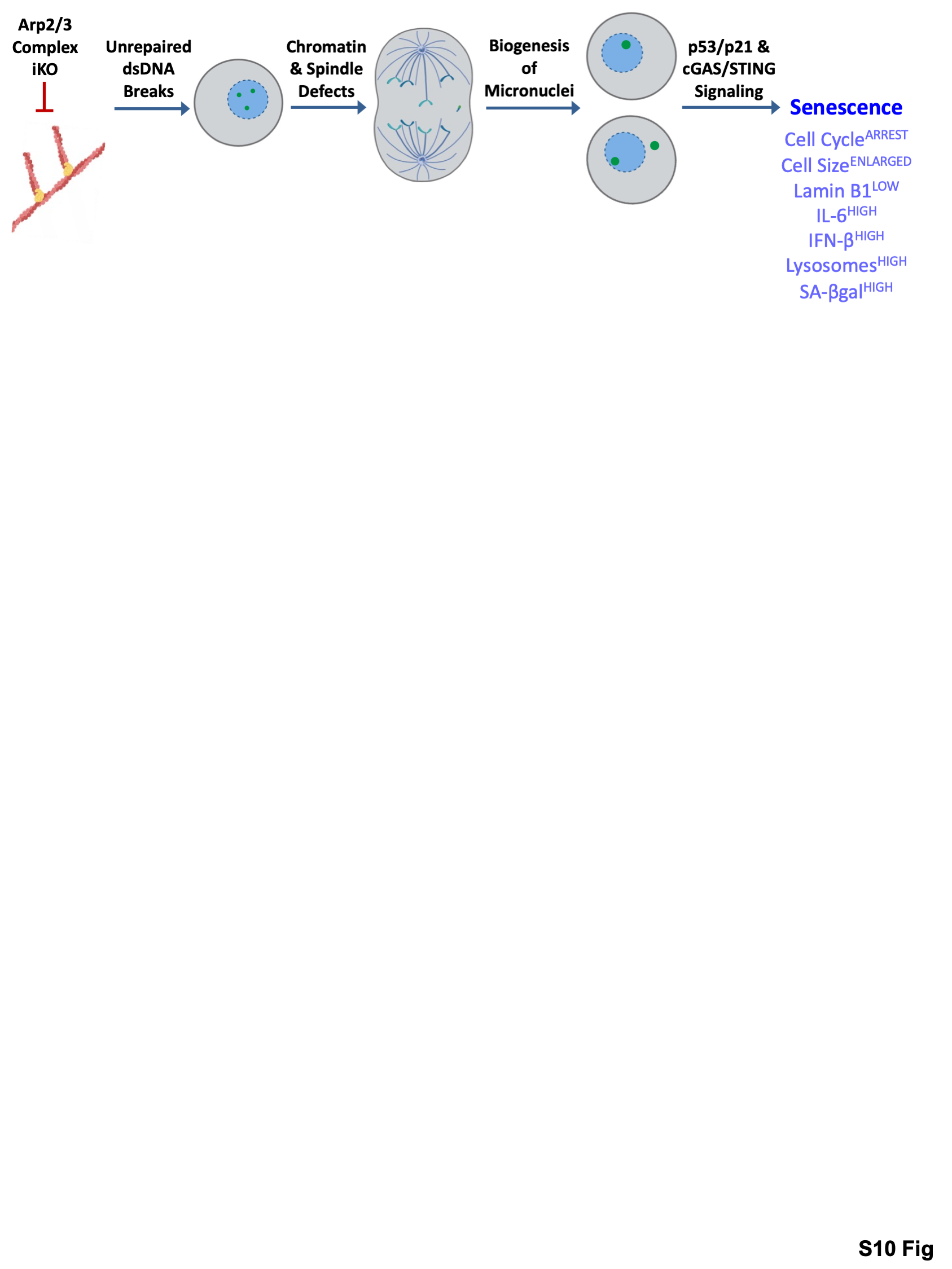

Supplement: S10 Fig — (TIF) [file pgen.1010045.s012.tif]
