# Supplementary material for: Genomic instability caused by Arp2/3 complex inactivation results in micronucleus biogenesis and cellular senescence
Source: PLoS Genet. 2023 Jan 27;19(1):e1010045. doi: 10.1371/journal.pgen.1010045 (PMC9907832; doi:10.1371/journal.pgen.1010045)
Supplement: Attachment — Submitted filename: Haarer_Coverletter2.pdf [file pgen.1010045.s021.pdf]

UCONN
UNIVERSITY OF CONNECTICUT

DEPARTMENT OF MOLECULAR AND CELL BIOLOGY
INSTITUTE FOR SYSTEMS GENOMICS

KENNETH G CAMPELLONE, PHD
ASSOCIATE PROFESSOR
67 NORTH EAGLEVILLE RD - RM 216
STORRS, CT 06269-3197

PHONE: (860) 486-3326 LAB: (860) 486-2497
EMAIL: KENNETH.CAMPELLONE@UCONN.EDU
WEB: HTTPS://CAMPELLONELAB.UCONN.EDU
TWITTER: @CAMPELLONELAB

November 22, 2022

Dear *PLOS Genetics* Editors,

Thank you for your review of our manuscript "***Genomic instability caused by Arp2/3 complex inactivation results in micronucleus biogenesis and cellular senescence***" by Haarer et al. In response to the reviewers' comments, we have attached our revised manuscript.

Our paper is broken down into 3 conceptual parts: [1] proving that Arp2/3 complex iKO cells undergo senescence using a rigorous set of biomarkers (Figs 1-3), [2] showing that the mechanism of micronucleus biogenesis is defective mitoses involving damaged DNA accompanied by cytoskeletal abnormalities (Figs 4-7), and [3] demonstrating that the mechanism of cell cycle arrest primarily relies on *Cdkn1a*/p21 with secondary contributions from cGAS-STING signaling (Figs 8-11). Our new data amount to 16 additional panels in the 11 main Figs and another 27 panels in 10 supporting Figs (totaling 43 new panels in a paper with a grand total of 88 data panels). The main conclusions are still that Arp2/3 is fundamentally important for maintaining genomic fidelity and that in its absence cells undergo senescence. As described below, the revised paper provides more information about the senescence-induction mechanisms that result from Arp2/3 inactivation.

Briefly, our changes include new data on [1] the importance of p53/p21 in a G1 cell cycle arrest that takes place in Arp2/3 complex iKO cells, [2] several control experiments related to the specificity of Arp2/3 inactivation and the prevalence of DNA damage and cell proliferation blocks in multiple cell lines, [3] a further characterization of cGAS/STING and interferon response phenotypes in iKO cells, and [4] a consolidation and reorganization of figures that includes several image improvements and text clarifications. Our point-by-point responses to each reviewer appear in blue text below. We were disappointed that Reviewer 3 performed such a superficial reading of our paper (they even admitted this multiple times). They provided very unfocused comments, in part based on their lack of familiarity with cellular senescence. We nevertheless responded to several of their relevant critiques. Two versions of the text are attached: one 'marked up' copy with additions depicted in red font and one 'clean' copy. To complete the experimental revisions, and with the approval of all previous authors (Haarer, Guo, Campellone), we added two new contributing authors (Theodore and Frier) to the revised paper. Overall, we feel that our work will influence audiences interested in cytoskeletal functions, DNA damage responses, cancer biology, and cellular senescence/aging.

We apologize for the delay in resubmitting and thank you for your patience in considering our revised paper for publication in *PLOS Genetics*.

Sincerely,

Ken Campellone

Dear Dr Campellone,

Thank you very much for submitting your Research Article entitled 'Genomic instability caused by Arp2/3 complex inactivation results in micronucleus biogenesis and cellular senescence' to PLOS Genetics.

The manuscript was fully evaluated at the editorial level and by three independent peer reviewers. The reviewers appreciated the attention to an important problem, but raised some substantial concerns about the current manuscript. Based on the reviews, we will not be able to accept this version of the manuscript, but we would be willing to review a much-revised version. We anticipate that the revision will require additional experimentation. We cannot, of course, promise publication at that time.

Should you decide to revise the manuscript for further consideration here, your revisions should address the specific points made by each reviewer. We will also require a detailed list of your responses to the review comments and a description of the changes you have made in the manuscript.

If you decide to revise the manuscript for further consideration at PLOS Genetics, please aim to resubmit within the next 60 days, unless it will take extra time to address the concerns of the reviewers, in which case we would appreciate an expected resubmission date by email to plosgenetics@plos.org.

If present, accompanying reviewer attachments are included with this email; please notify the journal office if any appear to be missing. They will also be available for download from the link below. You can use this link to log into the system when you are ready to submit a revised version, having first consulted our Submission Checklist.

To enhance the reproducibility of your results, we recommend that you deposit your laboratory protocols in protocols.io, where a protocol can be assigned its own identifier (DOI) such that it can be cited independently in the future. Additionally, PLOS ONE offers an option to publish peer-reviewed clinical study protocols. Read more information on sharing protocols at https://plos.org/protocols?utm_medium=editorial-email&utm_source=authorletters&utm_campaign=protocols

Please be aware that our data availability policy requires that all numerical data underlying graphs or summary statistics are included with the submission, and you will need to provide this upon resubmission if not already present. In addition, we do not permit the inclusion of phrases such as "data not shown" or "unpublished results" in manuscripts. All points should be backed up by data provided with the submission.

While revising your submission, please upload your figure files to the Preflight Analysis and Conversion Engine (PACE) digital diagnostic tool. PACE helps ensure that figures meet PLOS requirements. To use PACE, you must first register as a user. Then, login and navigate to the UPLOAD tab, where you will find detailed instructions on how to use the tool. If you encounter any issues or have any questions when using PACE, please email us at figures@plos.org.

PLOS has incorporated Similarity Check, powered by iThenticate, into its journal-wide submission system in order to screen submitted content for originality before publication. Each PLOS journal undertakes screening on a proportion of submitted articles. You will be contacted if needed following the screening process.

To resubmit, use the link below and 'Revise Submission' in the 'Submissions Needing Revision' folder.

We are sorry that we cannot be more positive about your manuscript at this stage. Please do not hesitate to contact us if you have any concerns or questions.

Yours sincerely,

Gregory P. Copenhaver, Ph.D.
Editor-in-Chief
PLOS Genetics

Gregory Barsh
Editor-in-Chief
PLOS Genetics

Reviewer #1: Elena L. Haare et al. showed in this manuscript that ArpC2 depletion induced cell cycle arrest with up-regulation of senescence markers such as p21/p53, DNA damage markers, and SAbetaGal staining in p16 knockout mouse tail fibroblasts. They also found that ArpC2 depletion resulted in the cGAS STING recruitment to the micronuclei and the formation of cytosolic micronuclei. These findings about the association between cytoskeletal dysfunction and cellular senescence are interesting. Using the several senescent markers and assays, the authors clearly showed that ArpC2 iKO cells became senescence like phenotype, however I have some concern about the molecular mechanisms and causality. Especially, the mechanisms about cGAS STING pathway and micronuclei formation are descriptive so far. Furthermore, they did all experiments in p16 knockout mouse tail fibroblasts. The following issues should be addressed to make the findings more robust and conclusive.

Major issue

1. In Fig 1A, why Arp3 expression was decreased in ArpC2 iKO cells? To confirm the specificity of ArpC2 iKO model, the protein and mRNA expression level of all seven subunits of Arp2/3 complex should be shown. The word in the title "Arp2/3 complex inactivation" is also obscure, not accurate because they only deplete ArpC2 and provide only ArpC2 and Arp3 expression. In addition, it was reported that single depletion of either ArpC2 or Arp2 in the Ink4a/Arf-/- IA32 cell line was not stable and the knockdown of both subunits were needed to produce a stable Arp2/3 knockdown (Cell. 2012 Mar 2;148(5):973-87.). Although ArpC2 iKO is different system from shRNA targeting ArpC2, the data which is not consistent with the previous data needs careful verification and mechanisms.

-- The ArpC2 subunit forms part of the core of the Arp2/3 complex (Gournier et al., 1999; two decades of structural studies). It is widely accepted that removing a core subunit of the complex results in loss of the entire complex at the protein level, as was shown in the paper that the Reviewer cites (Wu et al., *Cell*, 2012) and their follow-up paper which additionally monitored transcriptional expression (Wu et al., *J Cell Biol*, 2013). As we mentioned in our paper (p.7), loss of the complex has already been shown for the iKO cells used in our study (ArpC2, Arp2, and Arp3 blots in Rotty et al., *Dev Cell*, 2015). The standard in the field is to show the loss of the targeted subunit and, if possible, another subunit. For the analysis of second subunit, we demonstrated that once *Arpc2* was deleted, the Arp3 protein was also lost. What our paper adds is a more thorough kinetic analysis of the depletion of both ArpC2 and Arp3. For the paper that the reviewer cites, it is true that the single shRNA-mediated knockdowns of ArpC2 or Arp2 were not stable. But their 2xKD turned out to not be particularly stable either – that is why they developed the iKO system that we currently use (Rotty et al., *Dev Cell*, 2015). Lastly, this is a genetic inactivation of the Arp2/3 complex; in our revised paper, we add pharmacological inactivation as well (see next point). Thus, we believe that "inactivation" is appropriate.

2. All the experiments showed in this manuscript are done with p16 knockout mouse tail fibroblasts. The author mentioned about the previous study in line 117 of page 7, which showed knockdown of both the ArpC2 and Arp2 subunits are culturable in a p16Ink4a/Arf Ko cells, however in this paper the growth curve of Arp2 shRNA in wild-type and Arf-/- MEFs were showed (Cell. 2012 Mar 2;148(5):973-87.). They also described in the discussion that "ArpC2 iKO induce p16 independent cellular senescence". They should try

to show the senescence induction by knockdown of Arp2/3 using other tumor cell lines. If it works, it is more interesting to induce senescence to tumor cell.
-- Based on this reviewer's comment, we have taken a pharmacological approach to show that immortal mouse NIH3T3 fibroblasts, mouse B16-F1 melanoma cells, and human U2OS osteosarcoma cells all require the Arp2/3 complex for normal proliferation. Repeated treatments with the Arp2/3 complex inhibitor CK666 over a period of several days resulted in DNA damage and inhibited growth of all 3 cell lines. These data now appear in [S5 Fig and S6 Fig]. Because pharmacological inactivation of Arp2/3 is never complete and such inhibition is only temporary, we focused on these immediate/early changes, as it was not practical to assess the later features of senescence that emerge at >2-3 days.

3. In 154 of page 8, "apoptotic cell phenotypes were not observed in the iKO cell population". If they don't provide the data, this should be omitted.
-- In the revised paper, we added FACS analyses showing that the iKO cell population does not contain sub-G1 apoptotic cells. [Fig 1F-G and S1 Fig]. The statement is further supported by the data from all of the fluorescence imaging in the paper; apoptotic cell phenotypes (rounding, blebbing, nuclear condensation) were not present. The lack of apoptosis was expected based on one of our previous papers (King et al., *PLOS Gen*, 2021).

4. In Fig 2A and 2B, showing only IL-6 is not enough for the evidence of increase of SASP. IL-1beta, IL-1 alpha, TNF alpha, IL-8, IL-10, and GDF15 etc should be measured.
-- Our follow-up immunoblotting data suggests that IL-1-beta production, a cytokine present in only some SASPs, is not increased in iKO cells [S2 Fig]. In the revised paper, we also added RT-PCRs showing that Ifn-beta, a different cytokine, is upregulated in the iKO cells [Fig 2C and 2D]. This ties into a cGAS/STING/IRF3 signaling pathway described below. For a more comprehensive look at SASP gene expression in Arp2/3 complex-depleted cells, we refer the reader to (Wu et al., *J Cell Biol*, 2013). We leave the study of immunological aspects of Arp2/3 depletion, including SASP production and cytokine signaling, to others in the field (Wu et al., *J Cell Biol*, 2013; Rotty et al., *Dev Cell*, 2017; Ronzier et al., *Cells*, 2022). Our language about SASP in the Results and Discussion remains restrained and limited to describing the IL-6 and Ifn-beta data.

5. In Fig 6A, 7C, WB data should be with quantification data.
-- In the revised paper, we have added quantification of p53 expression levels [Fig 8B] and nuclear phospho-p53 levels [Fig 8E], as well as cGAS and STING expression levels [Fig 10C]. The abundance of p53 and P-p53 increases upon loss of the Arp2/3 complex, while cGAS and STING abundance is similar in Flox and iKO cells.

6. In Fig 7, the molecular mechanisms related with cGAS/STING signal and p53/p21 signals are descriptive. How cytoskeletal dysfunction activate cGAS/STING signals? The immunostaining data were not reliable at all. TBK1, NfkB, IRF3, and INFb expression etc should be analyzed.
-- As described in the paper, we show that micronuclei arise from damaged DNA and mitotic defects [Figs 4-7], and that these micronuclei recruit cGAS [Fig 10]. As mentioned in point #5 above and depicted in revised figures [Figs 8-9], the DNA damage caused by the loss of the Arp2/3 complex causes upregulation of p53 and p21 (RT-PCR, immunoblotting,

and/or immunofluorescence). In the revised paper we additionally demonstrate that RNAi-mediated suppression of p21 upregulation in iKO cells improves their mitotic index and proliferation, suggesting that iKO arrest is normally p21-dependent [Fig 9E-H]. After recognition of micronuclei, our data our consistent with previous observations of cGAS-STING signaling in cells with micronuclei (reviewed in Zhao et al., *Nat Rev Immunol*, 2022). It is unclear what the reviewer means by immunostaining data were "not reliable". Briefly, we show that tagged and endogenous cGAS are recruited to micronuclei and that STING is also in the area surrounding micronuclei [Fig 10A and 10D]. To address the Reviewer's comment, we have added several improved new images of ER-associated STING localization [Fig 10E]. In other new data, we show that phosphorylated (e.g., active) STING is at the Golgi in iKO cells unless cGAS is inhibited with RU.521 [Fig 11A]. Moreover, nuclear IRF3 levels are increased in iKO cells compared to Flox cells, but suppressed when cells are treated with RU.521 [Fig 11B and 11C]. The increase in Ifn-beta expression was also measured by RT-PCR, as mentioned above [Fig 2C].

7. In Fig 7F, validation data of RU.521 such as inhibition of downstream signals (NfkB, IRF3, and INFbeta) should be provided. Line 311 in page 14, If they mention "that cGAS inhibition has the potential to oppose the initiation of senescence in some of the cells in this experimental system. Collectively, the above localization and inhibitor results support the idea that cGAS/STING signaling contributes to the establishment of senescence in Arp2/3-deficient cells.", they should show the key data in Fig1-4, 6 using RU.521.
-- As mentioned in point #6, we added IRF3 nuclear localization data -/+ RU.521 (Fig 11B and 11C). We also added STING Golgi localization and phosphorylation data -/+ RU.521 (Fig 11A). As described in the paper and in point #6 above, we think that the major senescence induction comes from the DNA damage response (p53-p21) and that cGAS-STING signaling is secondary to that. Targeting cGAS via RU.521 slightly increases proliferation within the iKO cell population [Fig 11D]. In our hands, the drug is somewhat effective at inhibiting STING phosphorylation/relocalization and IRF3 upregulation and improving replication in a subset of cells if the drug is replenished frequently during the short-term assays [Fig 11]. But the drug is unlikely to be good enough for long-term (6-9 day) senescence studies, at least in our hands.

8. To show the robust causality, the "rescue" experiment, overexpression of ArpC2 in iKO cells should be considered.
-- We have added data showing that transient ArpC2-GFP rescue prevents DNA damage (gammaH2AX staining) in iKO cells [S7 Fig]. We also added data showing that induction of CreER in other (ArpC2-proficient) control cells does not cause DNA damage, micronucleus biogenesis, or prevent proliferation [S3 Fig].

9. Line 478 in page 21, to show that "ArpC2 iKO cells activate p53 and accumulate p21 in a p16- independent manner", the data using p53 inhibitor, commercially available, or using p53 mutated cell lines would be important.
-- Instead of targeting p53, we targeted p21 using RNAi, as described above in point #6 [Fig 9]. Prevention of p21 upregulation improved iKO cell proliferation. Activation of p53 (expression, nuclear localization, phosphorylation) is now shown more quantitatively in [Fig 8].

10. Are the expression of each Arp2/3 subunits increased in senescent cells?
-- Arp2/3 complex subunits are not expressed in ArpC2 iKO senescent cells. If the Reviewer is asking whether Arp2/3 is differentially expressed in other types of senescent cells (e.g., following telomere shortening, oncogene activation, mitochondrial function, etc.), the short answer is that we don't know. Some supplementary datasets from other investigators suggest that other regulators of the actin cytoskeleton might be increased, but Arp2/3 data are unclear. This would require extensive bioinformatic analyses that are beyond the focus of the current paper.

11. Regarding "impairment of Arp2/3 function in vivo could be a contributor to the development of age-related dysfunction and cancers" in line 511 of page 22, is there any evidence that the relationship between Arp2/3 mutation or SNPs and diseases?
In this manuscript, they showed only in vitro data using only p16 knockout mouse tail fibroblasts. That's a huge leap in logic.
-- Arp2/3 complex is dysregulated in cancers (reviewed in Molinie and Gautreau, *Physiol Rev*, 2018) and mutations are found in diseases (reviewed in Kramer et al., *Eur J Cell Biol*, 2022; Kahr et al., *Nat Commun*, 2017; Cintron et al., 2021; etc.). In the Discussion we are simply speculating about the potential importance of Arp2/3 in vivo.

Reviewer #2: The study by Haarer et al. focuses on the role of Arp2/3 during mitosis and the associated genomic integrity. The study is based on an elegant method for an inducible knock out (KO) of the ArpC2 subunit. The ArpC2 KO resulted in DNA damage, formation of cytosolic micronuclei and senescence. In the last part of the study the authors analyze changes in the organization of mitotic spindle actin and spindle microtubules.
As the authors mention in their discussion spindle defects can cause chromosome segregation mistakes and premature mitotic exits which can explain most of the phenotypes observed in ArpC2 KO cells (i.e. DNA damage, micronuclei and senescence). Indeed, previous studies using Arp2/3 inhibitors have revealed spindle- and chromosome segregation defects. It could be a point of criticism that the results are somehow expected from previous studies. However, the study is experimentally solid and gives a broad scope for the importance of Arp2/3 in mitosis and associated genomic stability. The focus on mitosis, DNA damage and senescence sets this study apart from many previous studies. Despite the sound experimental layout additional experiments and changes in the presentation should be made to increase the significance of the study.

Reviewer questions / suggestions for experiments and data presentation:
--Thanks for the suggestions.

1.) In Figure 1F an increase in cell area is shown and quantified in 1G. The difference is very robust in the field of view and quantification. This difference cannot be seen in Fig. 1C. Both images t=7d. Has Flox a different scale bar that was forgotten? The very uniform increase in cell- and nuclear area in Fig.1F (t=d7) is very heterogeneous in Fig. 2E (t=d9). Why?

-- As shown in previous Fig 1G (now Fig 1I), Flox and iKO populations include cells with an overlapping range of sizes (ranging from ~2000-8000 um2 in iKO cells). The ones in Fig 1C happen to be similar in size. The larger flatter cells are highlighted in Fig 1H, and heterogeneity in size is reflected in the image in Fig 2E (now 2F).

2.) In Figure 1F and G a clear increase in cell area and nuclear area is shown. The difference is so obvious that a flatter cell and nucleus are probably not the reason. Due to nuclear cytoplasmic scaling, this reviewer assumes that the cells are all already multiploid. This could be checked by a volume analysis and/ or FACS analysis for the DNA content (over time would be very good). This is interesting because here it looks like all cells (t=7d) had the same fate as the cells in Fig. 8B with premature mitotic exit. This could be the main phenotype leading to others like a senescent state. If this is true, it should be discussed. What is the ratio of cells with full KO t>5d complete mitosis vs. premature mitotic exit for example?
-- We're unsure about the consistency of nuclear-cytoplasmic scaling in this cell line/type, but certainly appreciate the suggestions and have performed FACS analyses for DNA content. The FACS data are shown in a new Fig 1F-G and S1 Fig. By 9 days, a G1 arrest is apparent, fewer cells are in S phase, and fewer are in "G2" as well [Fig 1F and 1G]. But as this reviewer smartly suggests, based on our movies in current Fig 5, the "G2" population contains many (mostly?) cells that underwent premature mitotic exits. A similar trend of G1 arrest and reduction in S was seen at 6 days [S1 Fig]. For the last point, unfortunately by >5d we are not able to catch any live mitoses – practically none of the iKO cells are mitotic [new Fig 9G]. In the Fig 5 movies, we have to view the cells using a 20x objective within the first 2 days of Arp2/3 depletion to capture significant numbers of mitoses. We believe that the sizes and shapes of the cells are in flux from days 2-10, as the senescent state develops and matures.

3.) In Fig. 2E magnifications should be provided that better show the phenotype to the reader.
-- To support the 20x images showing the breadth of Lamin B1 localization phenotypes, we have added 60x images of individual nuclei showing the primarily nucleoplasmic Lamin B1 localization in a Flox cell and its depletion in an iKO cell [Fig 2F]. (As a side note, Lamin B1 is mostly nucleoplasmic here and does not show a hardcore rimming of the nuclear envelope in interphase MTF cells like we see in other cell lines.)

4.) The graphical presentation of Fig. 2F could be improved. Individual datapoints seem to cover and hide each other. This can be avoided for superblots in the latest versions of programs like prism.
-- To show the distribution of Lamin B1 cellular phenotypes from independent experiments, those graphs include a lot of data points. Unfortunately, that causes a lot of point overlap. As a consequence, our graph resembles a violin plot more than a superplot, but it still illustrates the differences between Flox and iKO samples.

5.) In Fig. 3C+D images with higher resolution would help the reader. In Flox distinct lysosomes are visible. In KO the staining looks diffuse. However, it is likely a strong accumulation of lysosomes that just looks like a diffuse mass due to low resolution.

-- The Reviewer is correct in that the LysoTracker staining in Flox cells reveals typical-looking lysosomes and that LysoTracker staining in the iKO cells looks more like a diffuse mass. We describe the respective patterns as "discrete" and "punctate" versus "broad" and "diffuse". This means that 'images with higher resolution' are not available to be able to help the reader. The magnified images reflect the reality of diffuse LysoTracker staining in the Arp2/3 complex knockout cells. We think that this staining pattern is, at least in part, due to a lack of lysosomal membrane integrity/stability in the iKOs, and that formaldehyde fixation exacerbates a damaged or leaky lysosome phenotype. For the purpose of the current paper, our focus is on the fact that increased LysoTracker staining (acidic organelle content) is consistent with the independently-observed increase in SA-Bgal activity.

6.) Fig. 4A far right cell (KO) with micronuclei seems to be Fig.4B (vi) but mirrored. This reviewer has no issues with reusing this example in B. However, the mirror effect should be removed. If the authors agree, a sentence could tell the reader: vi is from Fig. 4A. If the authors see B as a fully independent figure, they might add to the legend of 4B: DNA was stained with DAPI. In general figure legends are rather short and could be revised that all information is given. As an alternative, phrases like "treated as in A" could be used.
-- We had flipped 4B(vi) to move the micronuclei away from the scale bar, but have flipped it back in the current version. We think we now have all the pertinent information in the Figure Legends, including general descriptions of the approaches, stainings, labels, and experimental and cellular n-values. For 11 main Figures, we have 7 pages of Legends. For more details, we also have 7 pages of Materials and Methods.

7.) In Fig. 6E seems to be a correlation between p21 intensity and nuclear size (multiploidy by mitosis defects? Please see point 2.) Is this true? Would it be interesting to analyze or discuss this?
-- Based on this interesting suggestion, we plotted nuclear area versus p21 intensity, but we did not see any positive correlation (slopes <0.1, R-squares <0.15). We think that p21 is upregulated in most cells irrespective of nuclear size, premature mitotic exits, or the presence of micronuclei, because ~all the cells undergo a DNA damage response. In the revised paper, we use RNAi to show that p21 upregulation is important for the proliferation arrest that normally takes place in ArpC2 iKO cells [Fig 9E-H].

8.) In Fig. 7D+E the authors mention a speckled ER-like localization of STING. In Fig. 7E this looks very randomly cytoplasmic. However, after zooming in, in Fig.7D the ER-localization seems to be nice and crisp. It appears feasible that the authors could provide a much better example in Fig. 7E.
-- In the revised paper, we keep the 20x images in Fig 10D to show general cGAS and STING localization in as many cells as possible at a low magnification. In Fig 10E, we replaced previous images with clearer higher magnification (60x) images of STING, GRP94 (ER), and GM130 (cis-Golgi). Also, in a new Fig 11A, we now show phosphorylated STING at the Golgi.

9.) In Fig. 8 the authors show nice mitosis defects. The figure layout appears narrow enough to add a magnification of the defect at the final timepoint. The defects were only visible after excessive zooming. A magnification will help the reader a lot going through the

figures. Related to that figure the authors provide video files. The submitted video files have a very poor quality compared to the still images in Fig. 8A+B. Can this problem be addressed by a better video conversion?
-- To show all the important panels in the timelapses, this figure (moved to Fig 5) is 9 images wide. The images are cropped to encompass both daughter nuclei at the end and nothing more. So we unfortunately cannot fit any further magnifications in this full-page figure and encourage zooming in the electronic images. We have re-saved the supplemental videos without compression.

10.) Fig. 9B shows quite nice the penetration of actin and microtubules in the metaphase plate. It would be helpful for the reader to have the same for KO cells.
-- In the revised paper, we have added an analogous image showing the lack of penetration in a mitotic iKO cell for direct comparison [S8 Fig].
In the low mag. images Fig. 9C it looks like the DNA containing region is more devoid of actin filaments and microtubule bundles. Quantification 9D shows that for actin but not significant for tubulin. A bulk fluorescence measurement in the chromatin area is not very robust for analyzing a complex structure like the spindle. However, the line scan 9E shows less prominent peaks for tubulin (microtubule bundles) than in Flox cells. The same is true for Fig. 10A+B. Also, the even microtubule distribution (symmetry) is affected as the authors mention.
-- That's right – microtubule density in metaphase looks a bit reduced, but the bulk difference did not reach statistical significance. Similarly, the linescans show modest differences for microtubules compared to more substantial ones for F-actin. At the request of another Reviewer, we measured F-actin and microtubules around centrosomes [S8 Fig]. There, no differences between Flox and iKO were detectable for microtubules or F-actin.
Arp-dependent actin polymerization seems to be necessary for proper microtubule spindle organization. Is this a take home message? Should it be discussed in more detail?
-- Based on the totality of our observations, our text explains that the presence of broken (presumably acentric) DNA fragments (e.g., Fig 5F, 6C, 7A) likely explains the formation of micronuclei in ArpC2 iKO cells. While the interesting lack of F-actin penetration into the central metaphase spindle and the uneven microtubule distribution in anaphase are likely direct and indirect consequences of Arp2/3 depletion, we don't want to overemphasize their importance because it is not yet possible for us to attribute these specific structural abnormalities to chromosome missegregation events. Therefore, we remain fairly conservative in our conclusions about Arp2/3 and spindle organization.

11.) The authors suggest that "in addition to the presence of misplaced damaged chromatin and a decrease in actin filaments at the metaphase plate, chromosome missegregations arising from alterations in anaphase microtubule organization MAY BE A CONTRIBUTING FACTOR in the formation of micronuclei in Arp2/3-deficient cells". Most micronuclei form during mitosis. ArpC2 KO causes spindle defects. This could lead to the hypothesis that Arp-dependent actin polymerization is important for spindle organization, KO of Arp causes spindle defects and mitosis errors which are the MAIN REASON for micronuclei, DNA-damage and senescence in this experimental setup. If mitosis is blocked / the cell cycle arrested… do KO cells accumulate DNA damage to a similar extent? If the block is

released, are they senescent or is senescence delayed until damage is acquired by additional mitosis?
-- Based on the literature showing the importance of Arp2/3 in DNA repair during interphase (Caridi et al., *Nature*, 2018; Schrank et al., *Nature*, 2018), and our observations of H2AX-positive foci in interphase and improperly localized H2AX-positive chromatin fragments in all stages of mitosis, we hypothesize that damage which takes place during DNA replication in S-phase and goes unrepaired due to Arp2/3-deficiency is responsible for the fragments that missegregate during mitosis. But we can't exclude the possibility that damage also takes place in mitosis. In either case, it likely goes unrepaired in interphase (e.g., Caridi, Schrank). We agree with the Reviewer that the spindle defects are important and that chromatin segregation errors are responsible for micronuclei. However, we do not have any clear evidence that spindle disorganization causes the DNA damage seen in iKO cells. Nor do we know the relative contributions of mitotic vs interphase damage. We think that a p21-mediated cell cycle arrest takes place during the G1 phase after a completed (2n cells) or aborted (4n cell) mitotic division. We appreciate the thoughts about experimentally blocking the cell cycle in other ways, but to coordinate those manipulations, troubleshoot, and unambiguously interpret those experiments was not something that we could do in the context of the current paper.

12.) Some experiments (without time course, most have a time course) were performed at different time points after induction of the KO. The authors should carefully clarify for the reader what was the ratio behind choosing different time points.
-- We have included rationales for different timings in the text.
Moreover, were certain phenotypes only visible at certain timepoints? For example, complete mitosis yielding micronuclei is a phenotype connected to partial depleted Arp functionality. Premature mitotic exit is prevailing at stronger Arp depletion. Are spindle defects more severe at d3-4 than on d1? The advantage of the system is that the authors can nicely correlate phenotypes to certain levels of depletion down to a full KO.
-- Each timepoint has a range of phenotypes that are generalized in the revised Discussion on p31. While the Reviewer nicely states an advantage of this experimental system, it does not allow us to know the % depletion in a particular live cell. By 3-4 days, mitoses are too infrequent to catch, especially when coupled with transient DNA transfections. So Fig 5 depicts the live cell phenotypes that are present within the first 2 days. We did not notice any differences in the frequencies of completed versus aborted mitoses on an hourly basis within that window.

13.) There are established ways to inhibit Arp2/3 functionality (CK-666, Arpin…). The reviewer is aware of the elegant system the authors use…Using a different way of inhibiting Arp2/3 functionality in 1 or 2 crucial experiments could further rule out the possibility of KO off target effects or effects by 4-OHT. Also 1-2 crucial experiments with an ArpC2 rescue by reexpression at an early timepoint, would be a valuable control of the results.
-- Yes, in the revised paper, we provide several additional controls. First, we added data indicating that induction of CreER in other (ArpC2-proficient) control cells does not cause DNA damage, micronucleus biogenesis, or prevent proliferation [S3 Fig]. Second, we have taken a pharmacological approach to show that immortal mouse NIH3T3 fibroblasts, mouse B16-F1 melanoma cells, and human U2OS osteosarcoma cells all require the Arp2/3

complex for normal proliferation. Repeated treatments with the Arp2/3 complex inhibitor CK666 over a period of several days resulted in H2AX-stained dsDNA breaks and inhibited growth of all 3 cell lines. These data now appear in [S5 Fig and S6 Fig]. Third, we have added data showing that transient ArpC2-GFP rescue prevents DNA damage (gammaH2AX staining) in ArpC2 iKO cells [S7 Fig].

Reviewer #3: In this work, the authors study effects of Arp2/3 complex inactivation on micronucleus biogenesis and the induction of cellular senescence. This is an exciting and very important topic, but unfortunately, the authors have chosen not to dig very deeply into the precise mechanistic reasons for the observations made. This also becomes clear when reading the discussion. I agree that the reasons for how Arp2/3 complex impacts on the these processes could be multiple, but this has already been evident before reading the article, so the question remains what precisely we can learn from the present study. It also seems a bit as if the authors have tried to pull on several ends simultaneously and have then thrown everything into one paper, but they failed, unfortunately, to come up with a conclusive story and key observations. Just as one example: the changes in lysotracker staining upon induced ArpC3-KO shown in Figure 3C are really dramatic, but what does this mean and how are these observations explained? As opposed to looking into this in more detail, the authors just moved on to the next observation, which makes the study quite descriptive. I do like the evidence that the micronuclei found in about 25% of the cells upon KO form during mitosis (and not other stages of the cell cycle), this is nicely demonstrated using video microscopy (Fig. 8), but the mechanistic reason for this is missing. I really have problems to believe in the described changes in actin filament penetration into the central spindle upon Arp2/3-KO or the described asymmetries in microtubule spindle organization (Figure 10). There are no convincing quantifications for the latter, and the data seem quite superficial. At this stage, I have just listed a few key points and problems that have come to my mind when reading this study.

1) Figure 1 is very thorough and nice, and should thus definitely been shown as is, although an increase in cell size upon acute Arp2/3 elimination was previously described in the literature (see e.g. PMID: 33598464), so this should at least be mentioned.
Aside from this though, I wondered: have the authors tried to clone out cell lines that escape from this suppression of proliferation? Importantly, a recent study described the generation of an HL60 cell line stably lacking Arp2 (PMID: 31600188, also used in 34096975), and I was disturbed by the fact that the authors did not even mention these recent studies published in in PLoS Biol and JCB. Later in the current work, the authors described genomic problems such as the formation of micronuclei in just a subfraction of cells (20% in Fig. 4C), so does this mean that cells not developing this phenotype could be convinced to continue proliferating, perhaps with conditioned media from wildtype cells? The authors are actually discussing paracrine phenomena as potential mechanisms in this context (see Discussion), so why was this not experimentally addressed?
-- We have added another citation of Dimchev et al., 2021 on p.10. As requested, we have also added citations using neutrophil-like cells from Graziano et al., 2019 and Pipathsouk et al., 2021 on p6. While the authors of those papers claim that the Arp2 cells were "null",

Arp2 protein expression is still clearly seen on the blots of the latter paper. We have not tried to clone out the escapees or suppressor mutants; while potentially interesting, such activities are clearly off-topic. In response to another suggestion, fresh media or media from WT cells does not 'convince' iKO cells to continue proliferating. Paracrine phenomena were not addressed because the paper focuses on the importance of a DNA damage response.

2) The observation in Fig 2 on the reduction in lamin B1 levels is very interesting, and likely important. It is also completely novel to my knowledge, but this is where the study ends, which is a shame. It would have been very interesting to address what the consequences of these reduced lamin B1 levels are – lamins have been previously connected to the development of progeria, correct? And it would have also been interesting to study here where this reduction in lamin B1 comes from – what is the mechanism of lamin B1 suppression? Can this be circumvented by ectopic expression? Can phenotypes of Arp2/3 KO described below be rescued by restoring lamin B1 levels?
-- As we stated in the text, reduction in Lamin B1 expression is a biomarker of cellular senescence identified about a decade ago (Freund et al., 2012), and is not 'completely novel'. Subsequently-published explanations for this phenotype are at the RNA and protein level, including transcriptional regulation, miRNA-associated regulation, changes in autophagy, etc. Hutchinson-Gilford progeria syndrome can be caused by mutations in the gene encoding Lamin A, not Lamin B1. Cells from such patients undergo premature senescence and some have reduced Lamin B1 levels. Elevations in Lamin B1 expression causes other diseases. While artificially increasing Lamin B1 levels will affect the chromatin landscape, it is not a logical approach for preventing senescence in our system. As mentioned elsewhere, since there is no singular marker for senescence, the purpose of the first 3 Figs is to use multiple well-established biomarkers to collectively prove that iKO cells are senescent.

3) The observations in Figure 3 are also interesting, but again, no follow-up experiments are performed. For instance, the changes in lysosome staining are stunning (Fig. 3C) and the consequences likely dramatic potentially, but without additional experiments, the results sort of stand alone without much context concerning specific Arp2/3 complex functions, which makes the study quite descriptive in places.
-- As we stated in the text, increased lysosomal content is another biomarker of senescent cells (Kurz et al., 2000; Lee et al., 2006). As mentioned above, since there is no singular marker for senescence, the purpose of the first 3 Figs is to use multiple well-established biomarkers to collectively prove that iKO cells are senescent. Fig 3 addresses SA-Bgal and acidic organelle abundance.

4) In general, Figures 2-5 display quite small datasets all together, so could be easily combined in one way or the other.
-- Fig 2 (now 7 panels) is about nuclear biomarkers and Fig 3 (4 panels) is about cytoplasmic biomarkers. They are conceptually distinct and are composed of too many panels to be combined, so we left them separate in the revision. However, the previous Figs 4-5 are better together, so we combined them into a single Fig 4 in the current paper, while moving part to S4 Fig.

5) The cGAS and STING stainings shown in Fig 7 are not as relevant in my view as some of the other observations, and could be easily moved to the Supplement perhaps. I also don't share the authors' view on the cGAS inhibitor experiments (RU.521). The conclusion by the authors was that RU.521 reduces the population doubling time upon Arp2/3 KO in a statistically significant fashion (Fig. 7F), but the effect was so tiny that I almost couldn't see it initially. I also wonder what sort of statistics was used for comparing these datasets, and how such minute changes could provide statistically significant differences (p-value of 0.02). I think looking at the Figure superficially, the conclusion could have just as well easily been that RU.521 does NOT show much of an effect at all.
-- Our statistical parameters are stated in the Methods and Legends. Fig 11D (formerly 7F) shows the means +/- SD from 5 experiments (t-test). When cells are cultured in parallel and subjected to robust titering, the population doubling times are highly reproducible. The iKO cell doubling time dropped from 25h to 23h. We describe this difference as "modest but statistically faster" in the text, because the difference is modest and statistically significant.

6) Figure 8 makes sense, but I was much less convinced with the observations described in Figs. 9 and 10. I really cannot discern a convincing, specific ArpC2-staining on or in the metaphase plate in Fig. 9A, so the statement (lines 380/1) that "these observations expand the catalog of F-actin and Arp2/3-associated cytoskeletal structures that are found within dividing mammalian cells" is certainly an over-statement.
-- We disagree. As does Reviewer 2 (explicitly) and Reviewer 1 (implicitly).
What about performing live-cell, confocal imaging of cells expressing EGFP-tagged Arp2/3 complex subunits? How about other Arp2/3 complex subunit antibodies? What do they show? There is no reason to restrict these stainings to just ArpC2. Are the authors sure the antibody is functional at all in IF? Can this be confirmed in interphase, control cells displaying lamellipodia?
-- In the revised paper, we added ArpC2-GFP images [S7 Fig]. The mouse anti-tubulin antibody cannot be used simultaneously with the mouse anti-Arp3 antibody. So we also added ArpC2/Arp3 double immunofluorescence [S8 Fig]. The antibodies were shown to be functional in IF in F-actin-rich ruffles and other structures (e.g., Fig 1; Velle and Campellone, *PLOS Pathogens*, 2018; Kabrawala et al., *PLOS Genetics*, 2020).

In addition, in Figure 9D, the quantification is concluded to show that actin filament intensities are reduced (which is perhaps the case), but microtubules are not. The reason for the latter conclusion appears to be the lack of statistical significance, but a trend of reduction is certainly observable as well, so here the drawn conclusions also seem to diverge quite significantly from the data shown (as above). If it holds true that removal of Arp2/3 reduces actin filaments in the metaphase plate during cell division, this should be studied and demonstrated using a variety of methods (including live cell, confocal imaging) much more thoroughly, because this then could be quite important, but the data as currently presented don't show this in a convincing fashion.
-- We are not inclined to draw conclusions based on a "trend of reduction". We and the other reviewers were convinced of the F-actin staining and quantification shown in our paper. The cytoskeletal abnormalities are an important, but relatively small, part of the

current paper, which is more about DNA damage, genomic instability, and senescence. We would like to visualize actin dynamics in live mitotic cells, but those experiments are very technically challenging, as evidenced by their infrequency in the literature (and the use of large oocytes for imaging such processes). Some of the technical challenges in visualizing F-actin in mitosis were mentioned elsewhere (Kita et al., 2019). We stand by our visualization of endogenous F-actin, ArpC2, and Arp3, especially compared to trying transient overexpression of fluorescent fusion proteins.

7) The potential misalignment of spindle microtubules in anaphase could be interesting as well (Figure 10), but again, the authors just show a very preliminary first dataset (basically one cell in each experimental group), without quantification how frequently this would occur, so again, the experiments sort of stop half way in between. Here, the question arises again where the potential mis-alignment comes from precisely? I guess it is difficult to exclude at least that this might also have to do with problems with centrosome function upon Arp2/3 depletion described by others previously, which is a possibility not even mentioned here. Although I am aware of the notion that spindles can self-assemble in principle in the absence of centrosomes, I am not sure whether one can exclude an impact of changes of actin assembly at centrosomes (caused by Arp2/3-KO) on the organization and dynamics of spindle microtubules, in particular in later stages of mitosis. Maybe I am wrong here and if this can really be excluded, but then it would be important to discuss it at least, and to perform more direct experiments to demonstrate how interference with Arp2/3 complex function during cell division mechanistically causes anaphase microtubule asymmetry and/or modified/abrograted spindle microtubule patterns and dynamics.
-- We didn't see any differences in centrosomal F-actin or microtubule staining. So in the revised paper we quantified those lack of differences [S8 Fig]. We also added more quantification of the left:right asymmetry in microtubule abundance [Fig 7C].
