# Supplementary material for: Genomic instability caused by Arp2/3 complex inactivation results in micronucleus biogenesis and cellular senescence
Source: PLoS Genet. 2023 Jan 27;19(1):e1010045. doi: 10.1371/journal.pgen.1010045 (PMC9907832; doi:10.1371/journal.pgen.1010045)
Supplement: Attachment — Submitted filename: Haarer_Coverletter+R2R_2023.pdf [file pgen.1010045.s022.pdf]

UCONN
UNIVERSITY OF CONNECTICUT

DEPARTMENT OF MOLECULAR AND CELL BIOLOGY
INSTITUTE FOR SYSTEMS GENOMICS

KENNETH G CAMPELLONE, PHD
ASSOCIATE PROFESSOR
67 NORTH EAGLEVILLE RD - RM 216
STORRS, CT 06269-3197

PHONE: (860) 486-3326 LAB: (860) 486-2497
EMAIL: KENNETH.CAMPELLONE@UCONN.EDU
WEB: HTTPS://CAMPELLONELAB.UCONN.EDU
TWITTER: @CAMPELLONELAB

January 6, 2023

Dear *PLOS Genetics* Editors,

Thank you for your review of our manuscript "***Genomic instability caused by Arp2/3 complex inactivation results in micronucleus biogenesis and cellular senescence***" by Haarer et al. In response to the reviewer's final comments, we have attached our revised manuscript.

After consultation with one of the editors, in this revision we have (i) made some text modifications, (ii) re-plotted some of the previous data (Fig 11C), and (iii) added new data on the nuclear-to-cytoplasmic ratio of the transcription factor IRF3 (Fig 11D). These additions clarify our cell size observations and imaging/quantification approaches. They also provide further support for our conclusions about cGAS-STING-IRF3 signaling. Our point-by-point responses to the reviewer appear in blue text below. Two versions of the text are attached: one 'marked up' copy with additions depicted in red font and one 'clean' copy.

Thank you again for considering our revised paper for publication in *PLOS Genetics*.

Sincerely,

Ken Campellone

Dear Dr Campellone,

Thank you very much for submitting your Research Article entitled 'Genomic instability caused by Arp2/3 complex inactivation results in micronucleus biogenesis and cellular senescence' to PLOS Genetics.

The manuscript was fully evaluated at the editorial level and by one of the original independent peer reviewers. The reviewer appreciated that you have made strides in revealing mechanistic insight, but have concerns that for these insights to be robust they need to be supported by quantitative analysis. Based on the reviews, we will not be able to accept this version of the manuscript, but we would be willing to review a much-revised version. We cannot, of course, promise publication at that time.
Should you decide to revise the manuscript for further consideration here, your revisions should address the specific points made by each reviewer. We will also require a detailed list of your responses to the review comments and a description of the changes you have made in the manuscript.
If you decide to revise the manuscript for further consideration at PLOS Genetics, please aim to resubmit within the next 60 days, unless it will take extra time to address the concerns of the reviewers, in which case we would appreciate an expected resubmission date by email to plosgenetics@plos.org.
If present, accompanying reviewer attachments are included with this email; please notify the journal office if any appear to be missing. They will also be available for download from the link below. You can use this link to log into the system when you are ready to submit a revised version, having first consulted our Submission Checklist.

To enhance the reproducibility of your results, we recommend that you deposit your laboratory protocols in protocols.io, where a protocol can be assigned its own identifier (DOI) such that it can be cited independently in the future. Additionally, PLOS ONE offers an option to publish peer-reviewed clinical study protocols. Read more information on sharing protocols at https://plos.org/protocols?utm_medium=editorial-email&utm_source=authorletters&utm_campaign=protocols
Please be aware that our data availability policy requires that all numerical data underlying graphs or summary statistics are included with the submission, and you will need to provide this upon resubmission if not already present. In addition, we do not permit the inclusion of phrases such as "data not shown" or "unpublished results" in manuscripts. All points should be backed up by data provided with the submission.
While revising your submission, please upload your figure files to the Preflight Analysis and Conversion Engine (PACE) digital diagnostic tool. PACE helps ensure that figures meet PLOS requirements. To use PACE, you must first register as a user. Then, login and navigate to the UPLOAD tab, where you will find detailed instructions on how to use the tool. If you encounter any issues or have any questions when using PACE, please email us at figures@plos.org.
PLOS has incorporated Similarity Check, powered by iThenticate, into its journal-wide submission system in order to screen submitted content for originality before publication. Each PLOS journal undertakes screening on a proportion of submitted articles. You will be contacted if needed following the screening process.

To resubmit, use the link below and 'Revise Submission' in the 'Submissions Needing Revision' folder.

We are sorry that we cannot be more positive about your manuscript at this stage. Please do not hesitate to contact us if you have any concerns or questions.

Yours sincerely,

Gregory P. Copenhaver
Editor-in-Chief
PLOS Genetics

Gregory Barsh
Editor-in-Chief
PLOS Genetics

Reviewer's Responses to Questions
Comments to the Authors:

Reviewer #1: Elena L. Haare et al. showed in this manuscript that ArpC2 depletion induced cell cycle arrest with up-regulation of senescence markers such as p21/p53, DNA damage markers, and SAbetaGal staining.

Some molecular mechanisms were shown in the revision manuscript. p21 senescent pathway was involved in the cytoskeletal dysfunction induced cellular senescence, and IRF3 high cells were significantly decreased by cGAS/STING signal inhibition. But quantification data of IRF3 >3 fold high cells are very subjective. Most of the data were shown as immunostaining data. Immunostaining is not useful tool for quantification in general. It is better to show a combination of other assays.
-- We appreciate the reviewer's interest in cGAS-STING-IRF3 signaling. But we respectfully disagree on the subjectivity/usefulness of immunofluorescence quantification. Fluorescence is inherently quantitative over a wide dynamic range and properly performed fluorescence microscopy provides very reliable quantitative information (e.g., Waters and Wittmann, *Methods Cell Biol*, 2014). Fluorescence, single cell analyses, and localization measurements (in our studies and in general) are more quantitative, sensitive, and informative than immunoblotting, RT-PCR, or other 'bulk' assays.

When we initially tested our antibodies to p53, P-p53, p21, cGAS, STING, phospho-STING, IRF3, and TBK1, we found that anti-p53 worked best in immunoblotting (Fig 8), anti-cGAS and -STING worked well both at immunoblotting and immunofluorescence (Fig 10), while anti-P-p53, -p21, -phospho-STING and -IRF3 were much more sensitive in immunofluorescence assays (Fig 8, 9, 11), and anti-TBK1 performed poorly in both types of assays (not shown).

To further support the robustness of our assays, in the current manuscript we revised and added new data to Fig 11. As before, our experiments yielded objective numerical values for relative protein levels. The cGAS-STING-IRF3 signaling phenotypes take place in subsets of cells, not the entire population. So to better illustrate this, we reformatted Fig 11C into (i) a dot plot and (ii) a bar graph of IRF^{HIGH} cells. To emphasize the IRF3 activation (nuclear import) phenotype, we now also demonstrate quantitative differences in the nuclear-to-cytoplasmic ratio of IRF3. This again utilized the very precise and highly reproducible analyses of single cell fluorescence. So in Fig 11D we present the IRF3 nuclear:cytoplasmic ratio (i) as a superplot and (ii) a bar graph of $IRF^{N>C}$ cells.

Overall, our readouts of cGAS activation and drug inhibition in Fig 11 are assessed by 3-4 different measures: phospho-STING relocalization to the Golgi, quantitative IRF3 accumulation in the nucleus (in 2 ways), and quantitative changes in cell proliferation. We believe that these data provide significant support for the presence of cGAS-STING regulation (which is secondary to both the cytoskeletal and p53-p21 phenotypes that we present) in Arp2/3 iKO cells.

In addition, as other reviewer mentioned, they showed that the cell area was increased in iKO cells, however, in Fig 1C, Fig 3C, Cig 8C, Fig 10D, and Fig 11B, cell size in iKO cells looks same as Flox cells.
-- We agree that the panels that the reviewer cites do include cells of similar size – this is not surprising because they fall within the overlapping ranges shown in Fig 1I. Flox cell sizes are typically in the 1000-3000um^2 range, while most iKO cell sizes are from 2000-6000um^2. Cell size is heterogeneous, and differences in Flox vs iKO cell size are more prevalent in Figs 1H, 2G, 3A, 4C top row, 8D, and 10D top row. We have added a sentence to the Materials and Methods (p.41) indicating that the distributions in MTF cell sizes shown in Fig 1I are reflected in the heterogeneity in cell sizes presented in later Figs.

I propose that the data about senescence markers should be in one figure, because a combination of

assays is needed to determine senescent cells. For example, cell cycle arrest (Fig 2D-G), beta gal (3A, B), p53(Fig8A-C), p21(Fig9A-C), LaminB1(Fig2E, F), and SASP markers (Fig2A-D).

-- We completely agree with the reviewer that a combination of assays is necessary to prove senescence. As we mentioned in the last coverletter (appreciating that the reviewer might not have had access to it), the assays and biomarkers in Figs 1-3 collectively show senescence – broken down as 1=arrest phenotypes, 2=nuclear phenotypes, 3=cytoplasmic phenotypes. We appreciate the reviewer's interest in consolidating the figures, and we drafted the 18-panel figure that they suggested (hypothetical Fig 2A-R), but squeezing the 18 panels of data into a single figure was not really feasible. It negated the general magnification requests from another reviewer, and caused circumvention of the journal's font size recommendations (8-12pt became 4-7pt and difficult to read). We considered several other figure panel rearrangements, but ended up with a 10-11 figure paper in each case. We therefore think that the current organization is the most straightforward for both figure and text organization.

Have all data underlying the figures and results presented in the manuscript been provided?

Large-scale datasets should be made available via a public repository as described in the *PLOS Genetics* data availability policy, and numerical data that underlies graphs or summary statistics should be provided in spreadsheet form as supporting information.

Reviewer #1: Yes
